# Gastric cancer surgery centralization under scrutiny

**DOI:** 10.1093/bjsopen/zraf185

**Published:** 2026-02-03

**Authors:** Styliani Mantziari, Maria Bencivenga, Ioannis Rouvelas

**Affiliations:** Department of Visceral Surgery, Lausanne University Hospital (CHUV), Lausanne, Switzerland; Faculty of Biology and Medicine, Lausanne University (UNIL), Lausanne, Switzerland; Department of Surgery, Dentistry, Paediatrics and Gynaecology—Esophageal and Gastric Cancer Surgery Division, University of Verona, Verona, Italy; Center for Digestive Diseases, Karolinska University Hospital and the Division of Surgery and Oncology, Department of Clinical Science Intervention and Technology (CLINTEC), Karolinska Institutet, Stockholm, Sweden

In the early 2000s, Birkmeyer *et al*. first reported an association between postoperative mortality and hospital surgical volume in the USA, laying the foundation for the volume-outcome paradigm^1^. This concept has since gained wide acceptance for complex procedures requiring technical expertise and specialized perioperative care. However, definitions of ‘high’ *versus* ‘low’ surgical volume centres vary considerably across countries, reflecting national demographics and disease prevalence. A recent systematic review supports the volume-outcome hypothesis for gastric cancer surgery, proposing a threshold of 100 gastrectomies per year being associated with lower mortality^2^. Yet this benchmark appears unrealistic for most Western countries, as most studies included in that review defined ‘high-volume’ centres within a range of 9 to 25 gastrectomies per year^2^.

In this issue of BJS Open, Gerber and colleagues explored the volume-outcome relationship in oncologic gastrectomy in Switzerland, where the Highly Specialized Medicine regulation governs several complex procedures such as pancreatic or oesophageal surgery, but not gastric resections^3^. Their study confirms that ‘high volume’, defined as more than 20 gastrectomies per year, consistent with Birkmeyer’s original threshold, was associated with a significantly lower postoperative mortality. Importantly, university hospitals and institutions with high patient volume reported reduced rates of failure-to-rescue in case of complications. Similar national evidence emerged from Italy, where data from the National Health Care Outcomes Program showed higher mortality after gastrectomy in low-volume compared with high-volume centres, as patients treated in high-volume hospitals were 26% less likely to die within 90 days^4^. In Sweden, Engborg *et al*. reported that centralization of gastric cancer surgery was associated with changes in treatment strategies and improved survival, reinforcing the broader European trend toward regionalized care^5^.

A multifaceted theoretical framework likely explains these observations. Rapid access to multidisciplinary expertise is critical for early identification and management of complications, thus reducing the risk of life-threatening deterioration. Moreover, structured morbidity-mortality audits not only help prevent avoidable complications but also create institutional reflexes that mitigate their impact when they do occur. While quality-improvement practices are not exclusive to high-volume centres, these institutions are more likely to have adequate structural capacity, resources and caseload needed to build and sustain institutional expertise.

Despite being a common malignancy worldwide, gastric cancer surgery often remains the ‘poor relative’ of complex oncologic procedures. A traditional argument has been that subtotal gastrectomy does not involve high mediastinal dissection or extensive lymphadenectomy. However, previous data from Japan also suggested a significant reduction in mortality for distal gastrectomy performed in high-volume centres (defined as >50 patients per year), with surgeon volume being less impactful^6^. Gerber *et al*. now confirm this finding in a Western population, showing a clear survival benefit for both total and partial gastrectomy^3^. Such improvements may reflect an ‘osmosis effect’, whereby expertise from other highly complex procedures such as oesophageal surgery enhances collective institutional performance across surgeons, anaesthesiologists, operating room personnel, and postoperative care units.

Although centralization may carry significant benefits in terms of postoperative outcomes, some potential pitfalls must also be acknowledged. Rather than focusing on volume alone, true performance should be captured through measures such as textbook outcomes, and quality assurance measured against international benchmarks. Concentrating oncologic surgery in a smaller number of reference centres needs investment in staffing, infrastructure, and operating capacity to avoid overwhelming high-volume institutions and prolonging patient waiting times. At the same time, caution is warranted to ensure that lower-volume centres with longstanding expertise and consistently good outcomes are not unjustly sidelined. Finally, the imposed burden, both financial and social, on patients who must travel further from home for specialized care, should be carefully considered. Clear referral pathways, well-functioning collaborations and networks, and transparent criteria for centralization are vital to maintain system sustainability while keeping patient safety and improved outcomes at the forefront.
